# A Contrastive Representation Learning Method for Event Classification in Φ-OTDR Systems

**DOI:** 10.3390/s25154744

**Published:** 2025-08-01

**Authors:** Tong Zhang, Xinjie Peng, Yifan Liu, Kaiyang Yin, Pengfei Li

**Affiliations:** School of Electrical and Mechanical Engineering, Pingdingshan University, Pingdingshan 467000, China; zhangtong@pdsu.edu.cn (T.Z.);

**Keywords:** Φ-OTDR systems, event classification, contrastive representation learning, wavelet transform convolution

## Abstract

The phase-sensitive optical time-domain reflectometry (Φ-OTDR) system has shown substantial potential in distributed acoustic sensing applications. Accurate event classification is crucial for effective deployment of Φ-OTDR systems, and various methods have been proposed for event classification in Φ-OTDR systems. However, most existing methods typically rely on sufficient labeled signal data for model training, which poses a major bottleneck in applying these methods due to the expensive and laborious process of labeling extensive data. To address this limitation, we propose CLWTNet, a novel contrastive representation learning method enhanced with wavelet transform convolution for event classification in Φ-OTDR systems. CLWTNet learns robust and discriminative representations directly from unlabeled signal data by transforming time-domain signals into STFT images and employing contrastive learning to maximize inter-class separation while preserving intra-class similarity. Furthermore, CLWTNet incorporates wavelet transform convolution to enhance its capacity to capture intricate features of event signals. The experimental results demonstrate that CLWTNet achieves competitive performance with the supervised representation learning methods and superior performance to unsupervised representation learning methods, even when training with unlabeled signal data. These findings highlight the effectiveness of CLWTNet in extracting discriminative representations without relying on labeled data, thereby enhancing data efficiency and reducing the costs and effort involved in extensive data labeling in practical Φ-OTDR system applications.

## 1. Introduction

Distributed optical fiber sensing systems utilize optical fibers as sensors to monitor objects, allowing for the identification and localization of various dynamic events occurring in the vicinity along fibers. The phase-sensitive optical time-domain reflectometry (Φ-OTDR) system is a distributed optical fiber sensing technology that utilizes the optical fiber itself as the sensing medium. Its operating principle relies on injecting highly coherent light pulses into the fiber by a narrow-linewidth laser. As these pulses propagate, they are scattered by the fiber’s natural, microscopic imperfections, which generates a stable Rayleigh-backscattered (RBS) light signal. When an external event, such as a vibration or acoustic wave, perturbs a section of the fiber, it induces localized changes in the fiber’s length and refractive index. This change modulates the phase of the RBS light returning from that specific location. By demodulating the backscattered light signal at the receiver, the system can detect, locate, and characterize the disturbance with high sensitivity and precision. This powerful capability has led to the system’s adoption in a wide range of applications, such as pipeline monitoring [1], perimeter security [2], structural health monitoring [3], and railway transportation monitoring [4]. However, in practical long-distance monitoring applications, the complexity of real-world environments, such as varying geological surroundings and high-intensity interference noise, often leads to false alarms and low event recognition rates [5]. Thus, emphasizing accuracy of event classification is crucial for effective deployment of Φ-OTDR sensing systems.

Over the past few years, researchers have been working on developing different methods to improve the accuracy of event classification in Φ-OTDR sensing systems. These methods generally fall into two categories: feature extraction methods and deep learning methods. The feature extraction methods extract important features from detected event signals by signal processing techniques, such as Fourier Transform [6], wavelet transform [7], Empirical Mode Decomposition [8], Principal Component Analysis (PCA) [9], joint analysis of the Relief F algorithm and correlation coefficient [10], and so on. Then, the extracted features are fed as the inputs to traditional machine learning classifiers, such as the Support Vector Machine (SVM) [11], Gaussian mixture model [12], and XGBoost [13]. Since detected signals depict essential characteristics of the event source, these feature extraction methods have achieved high classification accuracy. However, these methods typically require significant domain expertise to carefully select and extract meaningful features, and are sensitive to signal variations and the coherent-fading problem [14]. These limitations weaken the diversity and applicability of features derived from feature extraction methods, thus impeding their practical application in real-world scenarios.

Recently, the rapid development of deep learning has brought new opportunities for Φ-OTDR event classification, and some deep learning methods have been proposed to improve the accuracy and effectiveness of event classification [15]. Different from feature extraction methods, deep learning methods do not rely on feature extraction, but automatically learn representations from raw or minimally processed signal data collected by Φ-OTDR systems. For example, Shi et al. [14] proposed an event classification method that can directly learn representations from a collected temporal–spatial data matrix based on a CNN model. Wu et al. [16] utilized one-dimensional CNN to extract the distinguishable features from vibration signals for event classification. Sun et al. [17] adopted pulse scanning imaging to reconstruct the collected signals to form a two-dimensional image signal, and then trained a CNN network to classify the event signals. Wu et al. [18] combined 1DCNN with BiLSTM to extract the temporal structure information at each signal node and the spatial relationship among different signal nodes, thus improving the accuracy of event classification. In addition, Zhang et al. [19] proposed a multimodal feature fusion method that combines CNN and DenseNet to extract depth features from multiple angles and dimensions for accurate classification of event signals. Recently, Zeng et al. [20] developed a general method based on the Stockwell transform and STNet to effectively detect disturbances and accurately classify event signals in highly noisy environments. Le-Xuan et al. [21] proposed a hybrid deep learning model ResUNet4T by combining the strengths of U-Net and ResNet to improve the classification accuracy.

These deep learning methods have demonstrated high classification accuracy across different scenarios. However, most of them face a common constraint: they basically depend on a large number of accurately labeled samples for effective supervised model training. In Φ-OTDR systems, the significant reliance on labeled samples poses a major bottleneck in the application of deep learning methods for event classification. On the one hand, although event signals in Φ-OTDR systems can be detected and recorded by hardware devices and software, labeling a vast number of event signals is a time-consuming and labor-intensive task [22]. On the other hand, during the event signal collection process, a discrepancy exists between the event occurrence time and the signal labeling time. That is, when signals are manually labeled, there is a possibility that the event may have occurred some time ago, or it may not have occurred prior to the commencement of signal collection, ultimately leading to mislabeling of event signals [23]. These incorrectly labeled signal data will compromise the classification accuracy of these supervised deep learning methods.

Recently, some researchers have made attempts to alleviate the heavy dependence on a sufficient number of labeled samples to some extent. For example, Wang et al. [23] utilized the Siamese network to filter out the mislabeled signal data, which mitigates the problem of mislabeled data, and proposed a multichannel hybrid parallel classification network to learn representations from labeled signal data. Moreover, to alleviate the reliance on labeled training samples, Shilon et al. [24] employed the Generative Adversarial Net (GAN) to generate training datasets, and Zhang et al. [25] combined PCA with an unsupervised clustering algorithm (e.g., *k*-means) to classify events in Φ-OTDR systems. Recently, some studies have also explored transfer learning and few-shot learning methods for addressing scenarios where labeled samples for a specific event are scarce. For instance, Shi et al. [26] introduced a cycle generative adversarial network to augment the data amount of minor classes and retrained the pretrained AlexNet network for classifying different events. Subsequently, Shi et al. [22] proposed a hybrid approach for constructing training samples and a reinforcement learning method to recognize event types where labeled training samples were not available. Zhou et al. [27] utilized the time-shift strategy to augment the data, and integrated the U-Net structured network with the pretrained AlexNet to identify different event types. Although these methods have reduced the dependence on labeled training data to some extent, they still have some shortcomings. For instance, the classification accuracy still requires improvement, and there is also the issue of datasets shifting when transfer learning is conducted across different datasets.

With the rapid development of deep learning, contrastive representation learning has emerged as an effective method for addressing the issue of dependence on labeled data. Unlike traditional deep learning methods that rely on labeled data, the goal of contrastive representation learning is to learn useful and generalizable representations from existing unlabeled data [28,29]. It can generate supervisory labels from the data itself without relying on explicit labels. The supervisory labels include positive pairs, representing different views of a single data instance, and negative pairs, comprising views derived from different instances. Contrastive representation learning aims to learn an embedding space by maximizing the similarity between positive pairs while minimizing the similarity between negative pairs. In this space, positive samples are situated in close proximity, whereas negative samples are distantly separated. Consequently, contrastive representative learning empowers the model to capture the essential features and underlying similarities within the data, even in the absence of explicit labels. It has exhibited remarkable capabilities in learning meaningful representations for various types of data, such as time series and images [30]. This indicates its potential as a powerful tool for learning representations from unlabeled data in Φ-OTDR systems, presenting a promising way of overcoming the limitations of current supervised learning methods.

In this paper, we introduce CLWTNet, a novel contrastive representation learning method enhanced with wavelet transform convolution for event classification in Φ-OTDR systems. The main contribution of CLWTNet lies in its capacity to learn robust and discriminative representations directly from unlabeled Φ-OTDR signal data. In contrast to traditional supervised methods that rely on manually labeled datasets, CLWTNet aims to learn meaningful representations by leveraging the inherent structure and patterns embedded within unlabeled Φ-OTDR signals. By employing the principles of contrastive learning, CLWTNet acquires the ability to learn representations that draw similar Φ-OTDR signals closer together within the embedding space, while simultaneously pushing representations of dissimilar signals farther apart. Furthermore, CLWTNet incorporates wavelet transform convolution to enhance the extraction of multi-resolution time–frequency features inherent within Φ-OTDR signal data, which are crucial for discerning fine distinctions among various event types, thereby facilitating precision classification of different event types. CLWTNet possesses the capability to extract discriminative representations without the need for manual labeling, thereby enhancing data efficiency and mitigating the expenses and labor involved in extensive data labeling in practical applications of Φ-OTDR systems.

This paper is organized as follows: Section 2 details the methodology of CLWTNet. Section 3 describes the experimental data, evaluation metrics, and results of experiments conducted to evaluate the effectiveness of CLWTNet. Finally, Section 4 summarizes the conclusions of our paper.

## 2. Methodology

### 2.1. Overview of CLWTNet

**Figure 1:**
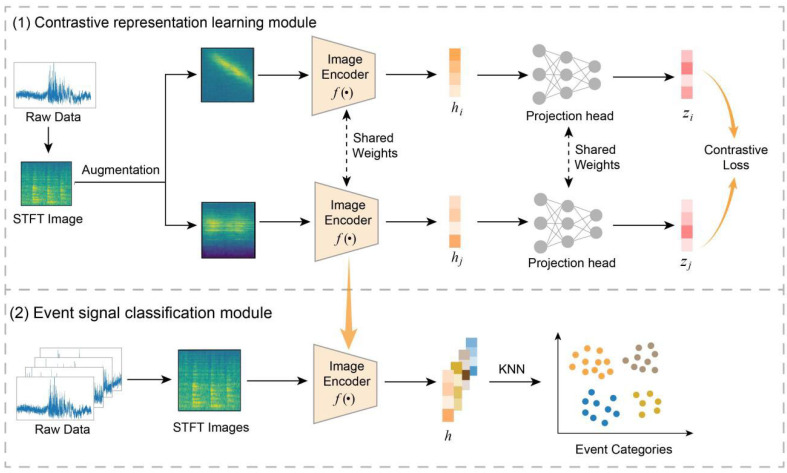
Overview of CLWTNet.

In this section, we introduce the proposed method, CLWTNet, which consists of two primary modules: the contrastive representation learning module and the event signal classification module. In the contrastive representation learning module, unlabeled raw event signals are initially transformed into STFT images via the Short-Time Fourier Transform (STFT). Then, an image encoder enhanced with wavelet transform convolution is trained to learn discriminative feature representations from the STFT image under the framework of contrastive learning. In the event signal classification module, the STFT images of event signals are fed into the trained image encoder to generate representations of each STFT image. Subsequently, the K-nearest neighbors (KNN) algorithm is used for unsupervised clustering of the feature representations, enabling the classification of event signals. The overall architecture of CLWTNet is illustrated in Figure 1.

### 2.2. Contrastive Representation Learning Module

The contrastive representation learning module is designed to learn meaningful representations from unlabeled signal data. Its main components consist of signal transformation, data augmentation, an image encoder, a projection head, and a contrastive loss function.

#### 2.2.1. Signal Transformation

The signal transformation process comprises two steps. Firstly, the one-dimensional signals collected by the Φ-OTDR system only reflect temporal fluctuations in the time domain, overlooking frequency variations over time. To address this, we employed the Short-Time Fourier Transform (STFT) to transform the one-dimensional time-domain signals into time–frequency images (i.e., STFT images). Specifically, we used a Hanning window to minimize spectral leakage, a window size of 64 samples, a 50% overlap (32 samples) to ensure that signal information at the window edges is not lost, and an FFT length of 64 points to match the window size. The STFT image size was fixed at 224 × 224 pixels. This image size not only aligns with the standard input dimensions used by prominent deep learning models such as ResNet and AlexNet, but also provides a good balance between feature detail and computational efficiency. Thus, the 224 × 224-pixel STFT images effectively preserve both temporal and spectral information, capturing the dynamic time-varying characteristics of the signal’s frequency components.

Secondly, despite the absence of labels for the event signals, we employed data augmentation to generate the necessary positive and negative samples for model training. Specifically, each STFT image undergoes augmentation through random cropping and resizing, color distortion, and Gaussian blur to produce two augmented views. Here, we adopted the augmentation parameters from the standard pipeline established in SimCLR [29]. Specifically, we performed a random crop of the original image, uniformly scaling its area between 0.08 and 1.0 while maintaining a random aspect ratio between 3/4 and 4/3. This cropped region was then resized back to the original size. An 80% probability color jitter transformation was applied, with brightness, contrast, and saturation factors set to 0.8 and a hue factor of 0.2. Additionally, we applied Gaussian blur with a 50% probability; its standard deviation was randomly selected from a uniform distribution ranging between 0.1 and 2.0.

In the training process, these two views, which are derived from the same STFT image, are considered as a positive pair. Conversely, other STFT images and their augmented views within the same training batch are treated as negative samples. Since the positive pair originates from the same STFT image, its members are expected to share similar features, whereas negative samples, derived from different STFT images, are more likely to contain distinct features. This data augmentation strategy can construct the necessary positive and negative samples for training without relying on explicit labels for the event signals.

#### 2.2.2. Image Encoder

**Figure 2:**
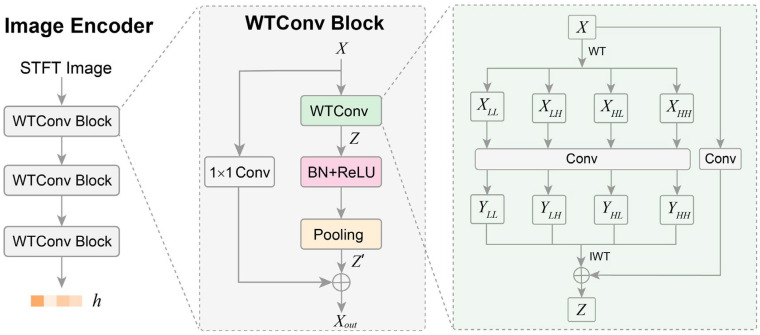
Structure of the image encoder.

The image encoder is designed to map the image of each augmented view into a feature space that effectively captures the underlying semantic content of the image. It plays a critical role in contrastive learning, as it determines the model’s ability to learn meaningful representations from the augmented views of STFT images. Here, we designed the image encoder enhanced with wavelet transform convolution (WTConv) to learn better representations. An overview of the structure of the image encoder is shown in Figure 2. It can be seen that the image encoder comprises three stacked WTConv blocks and takes the STFT image as input, subsequently outputting the corresponding representation *h*.

The WTConv block incorporates a wavelet transform convolution layer and residual connection to enhance the extraction of intrinsic features at different frequencies from the augmented-view images. In the WTConv block, the input *X* is first processed by a WTConv layer to generate an intermediate representation *Z*. Following batch normalization (BN) and ReLU activation, *Z* is then passed through a pooling layer to obtain *Z’*. Simultaneously, *X* undergoes a 1 × 1 convolution and is then added to *Z’* via a residual connection, resulting in the output *X_out_* of the WTConv block. Here, the 1 × 1 convolution projects *X* to a tensor to match the channel dimension of *Z’*. The WTConv layer is the key component of the WTConv block. In the WTConv layer, the input *X* is decomposed into a low-frequency component (*X_LL_*) and three high-frequency components (*X_LH_*, *X_HL_*, *X_HH_*) using the Haar wavelet transform, as shown in Equation (1). In these decomposition components, *X_LL_* contains a down-sampled, smoothed version of the original STFT image, preserving the large-scale structural information and overall shape of the image. *X_LH_*, *X_HL_*, and *X_HH_* capture the fine-grained, transient characteristics of the STFT image. For instance, *X_LH_* highlights sharp vertical edges in the STFT image, representing sudden events that are short in duration but span a wide range of frequencies. *X_HL_* captures sharp horizontal edges, representing persistent frequency components of the event signal. *X_HH_* detects high-frequency noise and fine textures of the STFT image. These decomposition components enable our WTConv layer to learn features tailored to both the stable, structural aspects and the sharp, transient aspects of event signals.

(1) $$X_{LL},\;X_{LH},\;X_{HL},\;X_{HH}=HaarWT\left(X\right)$$

(2) $$Y_{LL},\;Y_{LH},\;Y_{HL},\;Y_{HH}=Conv\left(X_{LL},\;X_{LH},\;X_{HL},\;X_{HH}\right)$$

(3) $$Z=IWT\left(Y_{LL},\;Y_{LH},\;Y_{HL},\;Y_{HH}\right)+Conv\left(X\right)$$

Next, a convolution layer with a 3 × 3 kernel is applied to *X_LL_*, *X_LH_*, *X_HL_*, and *X_HH_* to extract features at multiple frequencies, as described in Equation (2). Finally, the inverse wavelet transform (IWT) reconstructs the spatial features from *Y_LL_*, *Y_LH_*, *Y_HL_*, and *Y_HH_*. The reconstructed features are then added to the 1 × 1 convolution output of *X* to generate the intermediate representation *Z*, as described in Equation (3). The WTConv layer effectively captures multi-frequency spatial features without increasing substantial parameter overhead, thus providing meaningful representations of the image context [31].

#### 2.2.3. Projection Head

To facilitate the transformation of representation *h* into a low-dimensional space suitable for contrastive learning, we employ a projection head composed of a multi-layer perception (MLP). The MLP consists of three fully connected layers with 256, 512, and 128 neurons. The design of this MLP is guided by three purposes: (1) The 256-neuron input layer matches the dimensionality of the representation h from the image encoder. (2) The expansion to 512 neurons enhances the projection head’s nonlinear transformation capacity, allowing the model to learn a latent space where the contrastive loss function can be more effectively optimized. (3) The 128-neuron output is a commonly used and effective size for the embedding space where the contrastive loss is calculated, following the established contrastive learning framework [29]. Each layer is interleaved with batch normalization and ReLU activation. The projection head’s output, denoted as z, is then used in the contrastive loss function to measure the similarity among samples.

#### 2.2.4. Contrastive Loss Function

Here, we employ the normalized temperature-scaled cross-entropy loss [29], termed as NT-Xent, as the contrastive loss function to maximize the similarity between the learned representations of positive pairs while simultaneously minimizing the similarity with all other negative samples. Given a batch of *N* STFT images, two augmented views are generated for each STFT image, resulting in 2*N* data samples. Specifically, the two augmented views from the same STFT image are considered as positive samples, while all other 2(*N* − 1) data samples are treated as negative samples. Let $z_{i}$ and $z_{j}$ represent the outputs of the projection head derived from a positive pair of samples (*i*, *j*). The contrastive loss function $l\left(i,j\right)$ for samples (*i*, *j*) is defined as

(4) $$l\left(i,j\right)=-\log\frac{exp\left(sim\left(z_{i},\;z_{j}\right)/\tau \right)}{\sum_{k=1}^{2N}l_{\left[k\neq i\right]}exp\left(sim\left(z_{i},\;z_{k}\right)/\tau \right)}$$

where τ > 0 is a temperature parameter that controls the concentration level of the distribution, and $l_{\left[k\neq i\right]}\in \left\{0,1\right\}$ is an indicator function that evaluates to 1 if k≠i, and 0 otherwise. Here, we set τ = 0.5, following previous research [29].

The total loss for a batch is calculated by averaging all *N* pairs of positive samples, as defined in Equation (5).

(5) $$L=\frac{1}{2N}\sum_{k=1}^{N}\left[l\left(2k-1,2k\right)+l\left(2k,2k-1\right)\right]$$

During the training process, the weights of the image encoder and the projection head are updated to minimize the total loss L, thereby ensuring that the positive pairs of each STFT image are brought closer in the embedding space. Once the training process is completed, the well-trained image encoder can learn similar representations for positive pairs, while generating dissimilar representations between positive and negative samples.

### 2.3. Event Signal Classification Module

Here, we introduce the event signal classification module to classify event signals into distinct categories. As mentioned above, the well-trained image encoder can extract the intrinsic features of each STFT image, effectively bringing similar images closer and pushing dissimilar images farther apart. Therefore, the event signal classification module utilizes the representations generated by the well-trained image encoder to classify the event signals into distinct categories in an unsupervised manner. Specifically, the STFT images of event signals are fed into the trained image encoder to generate representations. These representations serve as high-dimensional embeddings of the STFT images within the learned feature space, effectively capturing the essential patterns that differentiate between STFT images of different event types.

Next, to classify the event signals, the KNN algorithm is employed to group the representations of STFT images into clusters that correspond to different event categories. Given an unknown event signal $s_{q}$ and its representation $h_{q}$ generated by the trained image encoder, the KNN algorithm computes the distances between $s_{q}$ and all training sample representations using the Euclidean distance. We assume that signals with the same event type typically share similar inherent features. Therefore, signals belonging to the same event type should exhibit minimal distances. Accordingly, we assign the unknown event signal $s_{q}$ to the class that is most common among its top *k* nearest neighbors, where *k* is a hyperparameter that determines the number of neighbors considered. Here, we set k=10. This *k* value is selected based on the consideration that large *k* values may oversmooth the KNN decision boundaries. Moreover, after an empirical search of the hyperparameter space, we determined that this *k* value can provide excellent classification accuracy for our task.

By combining contrastive representation learning with the KNN algorithm, CLWTNet presents a flexible method for classifying event signals in Φ-OTDR systems without relying on manually labeled signals for model training, enhancing its adaptability to real-world applications where labeled data is scarce.

## 3. Experiments and Results

### 3.1. Data Collection and Preprocessing

**Figure 3:**
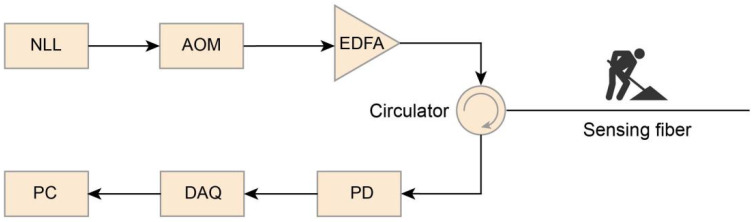
Diagram of the Φ-OTDR sensing system.

In this study, experimental data were collected using a home-made Φ-OTDR sensing system with a conventional setup similar to those used in previous studies [22,32,33]. As shown in Figure 3, an ultra-narrow-linewidth laser (NLL) generated the coherent optical pulses that were modulated into probe pluses by an acoustic optic modulator (AOM). The pulses were then amplified by an erbium-doped fiber amplifier (EDFA) and injected into a sensing fiber via a circulator. External events induced vibrations, leading to signal variations in the sensing fiber, which were captured by the Rayleigh-backscattered (RBS) light. The RBS light was collected by a photodetector (PD) and digitized using a data acquisition card (DAC) with a sampling frequency of 10 MHz to record changes in light intensity over time. Subsequently, the collected data were processed by a computer (PC).

In our experiment, different types of events were simulated near the sensing fiber, such as background, digging, knocking, watering, and shaking. To ensure consistent signal acquisition, each event was applied at a specific location along the fiber, and each collected event signal uniformly contained 10,000 time-domain points. For each type of event, we collected several hundred samples for the validation experiment. Table 1 provides the details of the five event types. We evaluated our CLWTNet and other baseline methods using a 5-fold cross-validation test. In the 5-fold cross-validation test, the entire dataset is divided into five non-overlapping folds of equal size. Each fold is used once as the test set, while the remaining four subsets serve as the training set. We performed five iterations of training and testing, ensuring that every sample was tested.

### 3.2. Evaluation Metrics

To demonstrate the effectiveness of our proposed method, CLWTNet, for event classification in Φ-OTDR systems, we utilized four metrics, namely accuracy, precision, recall, and F1-score, to compare its performance with other baselines. These four metrics are defined as Equations (6)–(9),

(6) $$accuracy=\frac{TP+TN}{TP+FP+TN+FN}$$

(7) $$precision=\frac{TP}{TP+FP}$$

(8) $$recall=\frac{TP}{TP+FN}$$

(9) $$F1-score=\frac{2\times precision\times recall}{precision+recall}$$

where *TP*, *FP*, *TN*, and *FN* represent the numbers of true positive, false positive, true negative, and false negative predictions, respectively.

Each of these metrics provides a unique perspective on the performance of the classification method. For example, accuracy offers an overall assessment of the method’s ability to correctly recognize different types of events in Φ-OTDR systems. Precision indicates the correctness of positive predictions, while recall assesses the method’s capacity to find all positive samples. F1-score provides a balanced evaluation of both precision and recall. Utilizing these four metrics, we conducted a comprehensive comparison experiment to evaluate the performance of our method.

### 3.3. Performance Comparison

To validate the effectiveness of our proposed method, CLWTNet, for event classification in Φ-OTDR systems, we conducted comprehensive comparisons against seven other methods. These seven methods are divided into three categories: (1) four unsupervised representation learning methods, including Autoencoder (AE) [34], Denoising Autoencoder (DAE) [35], Variational Autoencoder (VAE) [36], and a contrastive learning network (CLNet) [29]; (2) two supervised representation learning methods, namely ResNet [37] and AlexNet [38]; (3) a manual feature-based method named the MFM [11]. It is noteworthy that the four unsupervised representation learning methods, as well as our CLWTNet, are trained in an unsupervised manner, eliminating the need for labeled samples (i.e., signals with known event types). In contrast, CNN, MLP, and the MFM require labeled samples to learn representations. All methods were trained and tested with the same training and test datasets, as detailed in Table 2. We used a learning rate of 0.01, a batch size of 128, the NT-Xent contrastive loss function, and the Adam optimizer to train our CLWTNet model. All experiments were conducted on the Ubuntu server with an Intel Xeon CPU (2.4 GHz, 128 G RAM) and an Nvidia RTX 3090 GPU (24 G GPU RAM). The brief introductions of these methods are as follows:

- AE [34]: It performs encoding of an STFT image to a low-dimensional representation and then decoding of the low-dimensional representation to reconstruct the STFT image. The training process of AE is conducted in an unsupervised manner, without requiring labeled samples.
- DAE [35]: DAE is a variation of AE. Differently, DAE first introduces a corruption process applied to the STFT image and then reconstructs the original STFT image from low-dimensional representations, thus enhancing the robustness of representations learned from STFT images.
- VAE [36]: VAE incorporates probabilistic principles to map the STFT image to a set of probability distribution parameters in the latent space. Then, the latent representation is sampled from this distribution and used for reconstructing the STFT image. VAE is trained in an unsupervised manner by optimizing the reconstruction loss with label-free samples.
- CLNet [29]: CLNet is a contrastive representation learning method, and it is a variation of CLWTNet. Unlike CLWTNet, which employs WTConv layers, it uses CNN layers to construct the image encoder.
- ResNet [37]: This is a neural network architecture that utilizes residual connections to mitigate the vanishing gradient problem, allowing gradients to flow more effectively through the network during training. It is frequently used in image classification tasks.
- AlexNet [38]: This is a deeper convolutional neural network than ResNet, which includes multiple convolutional layers, ReLU activations, and dropout regularization. It has a profound influence in computer vision research.
- MFM [11]: A method is presented that manually extracts features from each event signal as representations for classification.

**Table 2:** Classification results of CLWTNet and other methods for classifying event types in the Φ-OTDR system.

Method Name	Accuracy	Precision	Recall	F1-Score
AE	0.774	0.769	0.768	0.767
DAE	0.787	0.786	0.781	0.781
VAE	0.798	0.795	0.789	0.789
CLNet	0.873	0.870	0.867	0.867
MFM	0.820	0.816	0.815	0.814
ResNet	0.925	0.922	0.920	0.919
AlexNet	0.924	0.919	0.918	0.917
CLWTNet	0.922	0.919	0.921	0.915

To compare the effectiveness of representations learned or extracted by different methods, the representations generated by each method were fed into the same classifier to evaluate the performance of each method. Here, we employed KNN as our classifier due to its simplicity, non-parametric nature, and the fact that it does not necessitate a training process. For an unclassified sample, KNN calculates its distance to all existing samples by using the Euclidean distance metric. Following this, the unclassified sample is assigned to the class that receives the majority vote from its top *k* nearest neighbors. We calculated the accuracy, precision, recall, and F1-score for each method, as shown in Table 2.

Firstly, we can see that the contrastive representation learning methods, namely CLNet and CLWTNet, exhibit superior performance in comparison to other unsupervised representation learning methods, such as AE, DAE, and VAE. For example, CLNet and CLWTNet achieve accuracies of 0.873 and 0.922, respectively, surpassing the accuracies of traditional autoencoder methods such as AE, DAE, and VAE, which record accuracies of 0.774, 0.787, and 0.798. These traditional autoencoder methods typically learn representations by reconstructing STFT images via encoder and decoder models. Differently, CLNet and CLWTNet utilize a contrastive representation learning module to extract meaningful representations from each STFT image by maximizing the agreement between augmented views of the same image. This result demonstrates the superiority of contrastive representation learning in extracting discriminative features from unlabeled STFT images, as compared to traditional autoencoder methods. Meanwhile, it is evident that our CLWTNet achieves better performance than CLNet. For example, the accuracy of CLWTNet (0.922) shows a notable improvement over the accuracy of CLNet (0.873). Unlike CLNet, which uses a CNN-based image encoder, our CLWTNet incorporates the WTConv block to construct its image encoder. Thus, this result highlights the effectiveness of the WTConv block in learning robust and discriminative representations from STFT images in an unsupervised manner.

Secondly, we find that the manual feature-based method (MFM) outperforms traditional autoencoder methods, including AE, DAE and VAE. However, it performs worse than contrastive representation learning methods, such as CLNet and CLWTConv, as well as supervised methods, such as ResNet and AlexNet. For example, the accuracy of the MFM is 0.820, surpassing the accuracies of AE (0.774), DAE (0.787), and VAE (0.798). However, it is inferior to CLNet (0.873), CLWTNet (0.922), ResNet (0.925), and AlexNet (0.924). These findings suggest that the learned representations, whether derived from supervised or contrastive learning methods, are capable of capturing more significant information than the predefined manual features used in the manual feature-based method.

**Figure 4:**
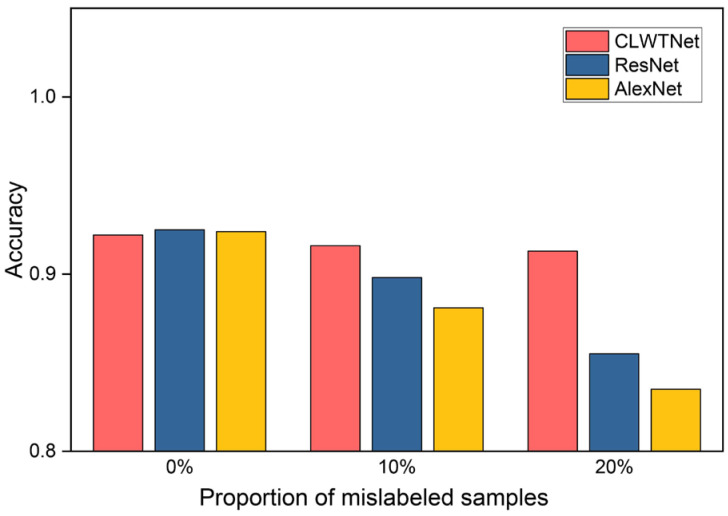
Accuracies of CLWTNet, ResNet, and AlexNet trained with 0%, 10%, and 20% mislabeled samples.

Thirdly, Table 2 reveals that the supervised representation learning methods AlexNet and ResNet achieve slightly higher accuracies (0.924 and 0.925) than our CLWTNet (0.922). However, this comparison result is obtained on a perfectly and accurately labeled training dataset. In the real-world Φ-OTDR sensing system, manually labeling signals may result in mislabeling of the collected signals. As reported in previous research, the proportion of mislabeled samples is at least 10% [23]. Since the supervised methods generally rely on sufficient samples with correct labels to achieve high performance, these mislabeled samples will degrade their performance. To assess the impact of mislabeled samples, we conducted a further comparison between CLWTNet, ResNet, and AlexNet, varying the proportion of mislabeled samples. Specifically, we incorporated 10% and 20% mislabeled samples into the training dataset, and trained CLWTNet, ResNet, and AlexNet accordingly. The accuracies of CLWTNet, ResNet, and AlexNet are illustrated in Figure 4. It is evident that the accuracies of ResNet and AlexNet decrease rapidly as the proportion of mislabeled samples increases, whereas the accuracies of CLWTNet remain almost consistent across varying proportions of mislabeled samples. These results highlight the effectiveness and applicability of our proposed contrastive representation learning method, particularly in real-world Φ-OTDR systems where samples are prone to mislabeling.

### 3.4. Analysis of Classification Performance

**Figure 5:**
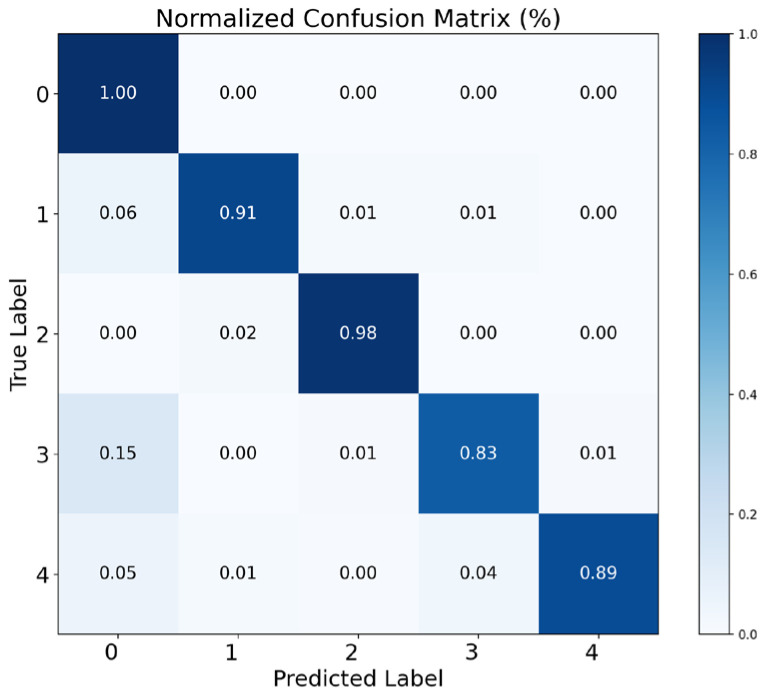
Normalized confusion matrix of CLWTNet for five events’ classification.

Furthermore, to investigate the classification ability of CLWTNet for different event types, we utilized the normalized confusion matrix to analyze the classification performance of CLWTNet for each event type. As shown in Figure 5, CLWTNet achieves near-perfect recognition for event types 0 and 2, with both precision and recall exceeding 0.98. The most challenging category is event 3, with 15% of samples incorrectly classified as event 0, and 2% of samples incorrectly classified as event 4 and event 2, respectively, resulting in a recall of 0.83. Moreover, the majority of residual errors come from confusion between event 0 and event 3, as well as between event 3 and event 4, suggesting the presence of semantic similarity among these event pairs.

**Figure 6:**
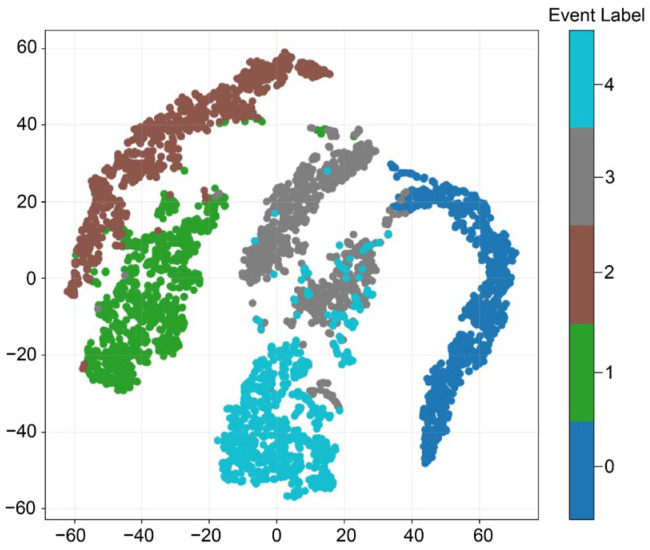
t-SNE visualization of representations learned from CLWTNet.

To visualize the distribution of representations learned by CLWTNet, we utilized the t-distributed stochastic neighbor embedding (t-SNE) method to project the learned representations into a two-dimensional apace. Specifically, we used the trained image encoder to generate 256-dimensional representations of STFT images. Then, t-SNE maps these representations into a two-dimensional space, wherein the representation of each sample is represented as a data point colored according to its event type. As shown in Figure 6, we can see that samples of different event types typically fall into distinct clusters. For example, samples of event types 0 (blue), 1 (green), and 2 (brown) form three clearly separated clusters. This suggests that the representations learned by CLWTNet are highly discriminative for these three event types of samples, enabling accurate classification for these samples. Meanwhile, samples of event types 3 (gray) and 4 (light blue) also form clear primary clusters, although some degree of overlap exists: for instance, in the region between the clusters of events 3 and 4, and the adjacency of clusters of event 3 and event 0. This result aligns with the discovery that the majority of residual errors in the confusion matrix come from confusion between event 0 and event 3, as well as between event 3 and event 4, as illustrated in Figure 5. This implies that CLWTNet not only captures discriminative representations that categorize different event types of samples but also learns their semantic similarities, thereby facilitating the effectiveness of event classification in Φ-OTDR systems.

## 4. Conclusions

In this study, we proposed CLWTNet, a novel contrastive representation learning method incorporating wavelet transform convolution, for robust and accurate event classification in Φ-OTDR systems. Furthermore, by transforming the original signals into STFT images, CLWTNet employs a specialized image encoder equipped with WTConv blocks to effectively capture multi-resolution time–frequency features essential for event classification. The experimental results showed that our CLWTNet, which was trained using unlabeled signal data, still achieves competitive performance compared to supervised representation learning methods, and significantly outperforms existing unsupervised representation learning techniques, thereby suggesting the effectiveness of CLWTNet in extracting discriminative representations without relying on labeled data. Notably, benefiting from not relying on labeled data for model training, CLWTNet maintains stable classification accuracy even in the presence of mislabeled samples, highlighting its robustness in real-world applications. Overall, CLWTNet presents a promising direction for enhancing the adaptability and effectiveness of event classification in Φ-OTDR systems, particularly in scenarios where labeling extensive data is expensive and laborious. Our future work will focus on expanding this method to adapt to multimodal data inputs, thereby further improving its accuracy and effectiveness.

## Figures and Tables

**Table 1:** The details of the five event types.

Event Type	Label	Number of Samples	Description
Background	0	547	The signals were collected during daytime and at night, in the absence of intentional interference.
Digging	1	793	A person used a shovel to dig near the sensing fiber at a rate of one second.
Knocking	2	890	A person used a shovel to tap near the sensing fiber at a rate of one second.
Watering	3	863	A person used a watering can to wash near the sensing fiber, positioned at a height of about half a meter.
Shaking	4	620	The fence equipped with the sensing fiber was vibrated by human movement to simulate climbing activities against the fence.

## Data Availability

This paper details the original contributions proposed in this study. For further inquiries, please direct your inquiries to the corresponding author.
